# Multiplexed Workplace Measurements in Biogas Plants Reveal Compositional Changes in Aerosol Properties

**DOI:** 10.1093/annweh/wxab036

**Published:** 2021-07-05

**Authors:** Dierk-Christoph Pöther, Daniela Schneider, Ulrich Prott, Jörg Karmann, Kerstin Klug, Nancy Heubach, Ralph Hebisch, Udo Jäckel

**Affiliations:** 1 Unit for Biological Agents, Federal Institute for Occupational Safety and Health, Nöldnerstr. 40–42, 10317 Berlin, Germany; 2 Unit for Measurement of Hazardous Substances, Federal Institute for Occupational Safety and Health, Friedrich-Henkel-Weg 1–25, 44149 Dortmund, Germany

**Keywords:** bioaerosol, clone library, endotoxin, hazardous substances, occupational health, risk assessment

## Abstract

Anaerobic digestion is an emerging technology producing energy from renewable resources or food waste. Exposure screenings, comprising hazardous substances and biological agents, at different workplaces are necessary for a comprehensive overview of potential hazards in order to assess the risk of employees in biogas plants. In order to analyse these parameters, workplace measurements were conducted in seven full-scale anaerobic digesters. Personal and stationary sampling was performed for inhalable and respirable particles, volatile organic compounds, ammonia, hydrogen sulphide, carbon monoxide, and carbon dioxide. Furthermore, concentrations of the total cell count, endotoxins, and fungi—down to species level—were determined in comparison to windward air. Sequencing of the 16S rRNA genes was utilized for the determination of the bacterial composition inside the biogas plants. Measurements of hazardous substances show hardly values reaching the specific occupational exposure limit value, except ammonia. An approximate 5-fold increase in the median of the total cell count, 15-fold in endotoxins, and 4-fold in fungi was monitored in the biogas plants compared with windward air. Specifying the comparison to selected workplaces showed the highest concentrations of these parameters for workplaces related to delivery and cleaning. Strikingly, the fungal composition drastically changed between windward air and burdened workplaces with an increase of *Aspergillus* species up to 250-fold and *Penicillium* species up to 400-fold. Sequence analyses of 16S rRNA genes revealed that many workplaces are dominated by the order of Bacillales or Lactobacillales, but many sequences were not assignable to known bacteria. Although significant changes inside the biogas plant compared with windward air were identified, that increase does not suggest stricter occupational safety measures at least when applying German policies. However, exposure to biological agents revealed wide ranges and specific workplace measurements should be conducted for risk assessment.

**What’s Important About This Paper?**

Biogas plants produce renewable energy through the anaerobic digestion and are increasing in number. This article provides a comprehensive aerosol exposure profile of workplaces in biogas plants. Although hazardous substances show no exceeded occupational exposure limits, except ammonia at a single setting, concentrations of biological agents in biogas plants are elevated and highly variable compared with windward air. This information is critical to understanding the health risks to workers in these emerging workplaces.

## Introduction

One of the major goals of humankind in the upcoming years is the increase of renewable energy in the global energy mix and eventually minimizing the threats of climate change (United Nations General Assembly, 2015). One pillar in the production of renewable energy is the anaerobic digestion and biomethanization in biogas plants, respectively. In biogas plants, various organic matter, e.g. animal manures, food waste, municipal organic solid waste, sewage sludge, or renewable raw material, is fermented anaerobically with the accompanying production of methane and carbon dioxide. In Europe, energy production of biogas plants reached 63.5 TWh in 2018 and 90 PJ of biomethane (European Biogas Association, 2019). Biomethane (8.2 PJ) was utilized in transport in Europe in 2018, whereas the USA utilized 30 PJ of biomethane in transport in 2019 (REN21 Secretariat, 2020). The number of biogas plants nearly tripled from ~6000 to ~18 000 from 2009 to 2018, but in the last 4 years, new establishments are slowing down (European Biogas Association, 2019). In Germany, about 9500 biogas plants are installed with an annual increase of about 150 between 2014 to 2018. In total, 46 000 people are employed in biogas plants in Germany (Fachverband Biogas, 2020).

Workplaces in biogas plants are often characterized by their close proximity to the organic matter, high wetness of the material but not necessarily high humidity and moderate temperatures in combination with disintegration and rearrangement of organic matter (Wisniewska *et al.*, 2020). These circumstances favour microbial growth and formation of bioaerosols and hazardous substances. Thus, threats due to hazardous substances and biological agents should be included in the risk assessment of these workplaces.

Traversi *et al*. (2015, 2018) analysed the composition of bioaerosols in biogas plants for specific bacteria, the fungal load, fractions of particulate matter (PM), or endotoxins in a PM-dependent manner. The culture-dependent analysis of specific bacteria revealed a huge quantitative variance in different biogas plants by several orders of magnitude accompanied by changes in the composition. PM and endotoxin analyses showed no alarming results in comparison to amounts in rural air. Dubuis *et al*. utilized next-generation sequencing in order to describe the bacterial composition in two biogas plants. Furthermore, moulds and endotoxins were quantified, and quantitative polymerase chain reaction (qPCR) was employed to detect specific bacteria (Dubuis *et al.*, 2017). The load of culturable moulds was higher in summer than in winter but did not exceed 5 × 10^4^ cfus m^−3^. Detection of specific bacteria by qPCR solely revealed high numbers for *Mycobacterium* (Dubuis *et al.*, 2017).

Measurements of hazardous substances from biogas plants mainly focussed on emissions of the plant rather than workplace measurements. Liebetrau *et al.* (2013) showed elevated methane concentrations at the digestate storage and at the gas processing units, i.e. combustion or upgrading. Similar results were detected by Fredenslund who found highest methane concentrations at digestate storage, gas outlets, and gas engines (Fredenslund *et al.*, 2018). Kuo and Dow (2017) analysed at the emission level the emissions from the combustions and found no elevated concentrations, except for NO_*x*_.

Currently, no occupational exposure limit values (OELV) are stated for biological agents or their derivatives, except an exposure limit for endotoxins of 90 EU m^−3^ in the Netherlands (Health Council of the Netherlands, 2010). For hazardous substances, a multiplicity of OELVs are stated with some differences between countries as extracted from GESTIS (Deutsche Gesetzliche Unfallversicherung, 2020): in Germany, there is a general threshold limit for the inhalable particle fraction at 10 mg m^−3^ and for the respirable particle fraction at 1.25 mg m^−3^. For ammonia, OELVs range from 7 mg m^−3^ in France to 35 mg m^−3^ as stated by the OSHA in the USA. In the Netherlands, the OELV for hydrogen sulphide is 2.3 mg m^−3^, whereas the highest limit is 14 mg m^−3^ in Australia, Hungary, Singapore, and South Korea. Carbon monoxide OELVs range from 20 mg m^−3^ in China up to 57 mg m^−3^ in Japan, whereas carbon dioxide has an OELV of ~9000 mg m^−3^ in almost every country listed in GESTIS.

Up to now, no study comprised an approach of measuring hazardous substances and biological agents at workplaces in biogas plants in parallel that can support risk assessment comprehensively. Furthermore, Traversi *et al.* (2018) clearly specified necessary analyses of workplace bioaerosols in biogas plants: in-depth analysis of fungi and viable but not culturable (VBNC) microorganisms.

Thus, the aim of our study was to analyse hazardous substances and biological agents in aerosols with the main focus on comparing different workplaces in biogas plants with the windward air and broaden the current knowledge with respect to the composition of the fungal load and VBNC microorganisms.

## Materials and methods

### Biogas plants and general sampling information

Samples were drawn in seven biogas plants across Germany utilizing different sources for biogas production (see Supplementary Tables S1 and S2, available at *Annals of Work Exposures and Health* online). One of them converted renewable raw material; the others used animal manures, food waste, or municipal waste. Specific characteristics of the biogas plants and the different workplace measurements are listed in Supplementary Tables S1 and S2 (available at *Annals of Work Exposures and Health* online). At location 1, no data of hazardous substances were acquired. Sampling was solely conducted at outside temperatures above 0°C and without rainfall as these meteorological parameters could interfere with the subsequent analyses. Personal air sampling was carried out during the activity of workers. Consequently, the duration of sampling varied depending on the kind and duration of activity under investigation. When stationary sampling took place, the sampling was 1.5 m above ground level. Positioning of the sampling system was as near as possible to the investigated workplace or activity. All determined values with the specific sampling volume and duration are listed in Supplementary Table S3 (available at *Annals of Work Exposures and Health* online).

### Analyses of hazardous substances

#### Measurements of NH_3_, CO, CO_2_, H_2_S, and volatile organic compounds

NH_3_, CO, CO_2_, H_2_S, and volatile organic compounds (VOCs) were measured using the direct reading instrument G460 (GfG GmbH, Dortmund, Germany) in diffusion mode and analysed electrochemically (NH_3_, H_2_S, and CO), infrared detection (CO_2_), or by photo ionization detection (VOCs) with following measuring ranges: NH_3_ 0–200 ppm, CO 0–500 ppm, CO_2_ 0–5% (v/v), H_2_S 0–100 ppm, and VOCs 0–500 ppm. Additionally, VOCs were collected by the personal air sampler Gilian LFS-133 DC (Sensidyne LP, St. Petersburg, FL), with 10 ml min^−1^ onto thermal desorption tubes using Chromosorb 106 as sorbent. Subsequent analysis was carried out after thermal desorption using a Turbo Matrix 650 coupled with gas chromatography utilizing a Clarus 680 with flame ionization detection (ATD+GC/FID, Perkin Elmer GC, Shelton, CT).

#### Measurement of inhalable and respirable particle fractions, cobalt, and selenium

For personal and stationary sampling, airborne particles were collected with a flow rate of 10 l min^−1^ using personal air samplers SG10-2 (GSA GmbH, Ratingen, Germany) equipped with FSP10 and GSP10 sampling heads for respirable and inhalable particles, respectively. Quartz fibre filters or cellulose nitrate filters with 37 mm diameter were used in both approaches.

Additionally, respirable particles were collected with a flow rate of 46.7 l min^−1^ using the stationary sampler MPG III (Dr-Ing. Wazau, Berlin, Germany) connected to an Amicus pump (Leschke Messtechnik GmbH, Frankfurt/Oder, Germany) equipped with quartz or glass fibre filters with 47 mm diameter (e.g. MN QF10, Macherey Nagel, Düren, Germany). For inhalable particles, the stationary sampler Gravikon VC25 (Ströhlein Instruments, Kaarst, Germany) was utilized with a flow rate of 22.5 m^3^ h^−1^ equipped with, e.g., quartz fibre filters with 150 mm diameter (Whatman International Lrd, Maidstone, UK). For each instrument used, specific sampling heads were utilized which sample the particle fraction in accordance with the separation characteristics described in EN 481 (DIN/CEN, 1993).

Inhalable and respirable particles were analysed gravimetrically, whereas selenium and cobalt were extracted from filters by a mixture of hydrochloric acid and nitric acid (~1:2 v/v) (Institute for Occupational Safety of the German Social Accident Insurance, 2013) and analysed by atomic absorption spectrometry using AA240Z (Varian Inc, Mulgarve, Australia), with graphite furnace atomization and Zeeman background correction.

### Analyses of biological agents

#### Collection of bioaerosols

For personal sampling, inhalable bioaerosols were collected by personal air samplers SKC or GilAir with a flow rate of 3.5 l min^−1^. Polycarbonate filters were employed for the determination of the total cell count and glass fibre filters for determining concentrations of endotoxins. Sampling of total cell count (TCC) and endotoxins was carried out simultaneously. Furthermore, bioaerosols were sampled by the stationary Holbach air sampler collecting at a flow rate of 30 l min^−1^ either onto polycarbonate filters for generation of 16S rRNA gene clone libraries or onto gelatine filters for determination of culturable airborne moulds.

Windward air samples were taken at the windward side of the biogas plant ~250 m away from the biogas plant in parallel to the other samples with the same instrumentation.

#### Determination of total cell count

TCC was determined as described by Martin *et al.* (2010). In brief, bioaerosols were mechanically detached from filters and suspended into physiological saline by a Stomacher. Afterwards, the suspension was fixed by formaldehyde, stained with 4′,6-diamidin-2-phenylindol, and filtered. Fluorescently labelled cells on defined regions of the filters were counted, and TCC m^−3^ was determined using the known sampling volume.

#### Determination of moulds

Quantification and identification of moulds was conducted as described elsewhere (Klug *et al.*, 2011). In brief, bioaerosol loaded gelatine filters were dissolved in physiological saline at 40°C. Serial 10-fold dilutions were plated in triplicates onto either malt extract or DG18 agar and incubated at 25 and 37°C, respectively. Mould colonies were counted and morphologically characterized after at least 4 days by the health department of the state Baden Württemberg and Umweltmykologie GmbH, Berlin, Germany, respectively. If the same fungus grew at different growth conditions, higher cfu values were chosen for further analysis as better growth conditions were assumed. Nonetheless, cfu ratios between these conditions were mostly below a factor of 2 for different culture media.

#### Determination of endotoxins

Endotoxins were determined as described by the national standard (Institute for Occupational Safety of the German Social Accident Insurance, 2002). In brief, filters were extracted in 10 ml pyrogen-free water (ACC Europe GmbH, Mörfelden-Walldorf) by shaking for 1 h on an orbital shaker at room temperature. The extract was centrifuged at 1000 g for 10 min. The analysis of endotoxins in the supernatant was performed (in duplicate) immediately after extraction using the kinetic chromogenic Limulus amoebocyte lysate assay (LAL) (Pyrochrome, ACC Europe GmbH, Mörfelden-Walldorf, Germany) according to the manufacturer’s instructions and analysed in a Biotek ELx808 with Gen5 software 3.08 (Biotek, Winooski, VT). Fifty microlitres of the supernatant were employed in the LAL assay.

#### Determination of bacterial composition using 16S rRNA gene clone libraries

The utilization of clone libraries for determination of bacterial composition was described by Martin *et al.* (2010). In brief, bioaerosols were mechanically detached from filters and suspended into physiological saline by a Stomacher and cells concentrated by centrifugation. DNA from cells was extracted using the GenElute Plant Genomic DNA Miniprep Kit following the manufacturer’s instructions, but an initial homogenization step was introduced by vortexing the suspension and the lysis buffer together with 0.5 g Zirkonia beads (diameter: 0.1 mm). DNA was quantified by Qubit dsDNA Broad Range Kit. Subsequently, the 16S rRNA gene was amplified using Primers established by Weisburg *et al.* (1991) covering nearly the whole 16S rRNA gene (43–1547 bp in *Escherichia coli* NCTC9001^T^) and amplicons were sent to LGC Genomics GmbH, Berlin, Germany, for construction of clone libraries. In total, the 16S rRNA gene inserts of 100 randomly picked clones were sequenced for each sample. Sequences were compared to GenBank using BLAST, and evolutionary distances were calculated using a Maximum Composite Likelihood model in order to identify closest relatives using MEGA 5.0 (Tamura *et al.*, 2011).

### Data analysis and visualization

Raw data were collected in Microsoft Excel. Data analysis and visualization were performed using R (3.6.1) and R-Studio (1.2.1335) with following packages: car (3.0–6), DescTools (0.99.32), dplyr (0.8.3), ggplot2 (3.2.1), ggpubr (0.2.3), ggrepel (0.8.1), ggsignif (0.6.0), gridExtra (2.3), RColorBrewer (1.1–2), and SysbioTreemaps (0.1.0). For statistical analyses between windward air and biogas plant, the Wilcoxon test was used as samples were not distributed normally (Shapiro-Wilk test) and were heteroscedastic (Levene test). The same is true for the tested workplace categories and thus the Kruskal–Wallis test with a *post hoc* Dunn test was chosen.

Voronoi Treemaps were calculated from the median of the identified fungal species or bacterial genera for the windward air samples and biogas plant samples, respectively. Via tessellation Voronoi Treemaps aim to completely fill the area with the given values for each level, i.e. species, genus, order, or class. Thus, the depicted area of a genus in the treemap represents the amount of the analysed genus in the sample. As an example species A, B, and C belong to genus X. Cfus of species A represent 2% of all cfu, species B 4%, and species C 10%. The utilized area of these species in the treemap will be 2, 4, and 10%. The utilized area of genus X is the sum of species A, B, and C, i.e. 16%. When comparing two treemaps as in Fig. 4, the sum of all cfus determines the total area of each treemap.

## Results

The main focus was the comparison of different workplaces. Thus, the sampling points were artificially categorized into the following workplace categories: inspection (perambulating in the facility and checking different machines), delivery (delivery of the fermentation sources outside or in a hall mainly in a bulldozer), sorting (sorting out unwanted material like stones; sorting and relocating waste; manually or inside a bulldozer), and cleaning (cleaning containers, tubes; desanding; often using high pressure cleaners). For biological agents, no OELVs exist. Thus, samples of the different workplace categories were compared with windward air in order to be able to analyse significant differences between regular and occupational exposition.

### Exposure to hazardous substances dominated by ammonia

**Figure 1.**
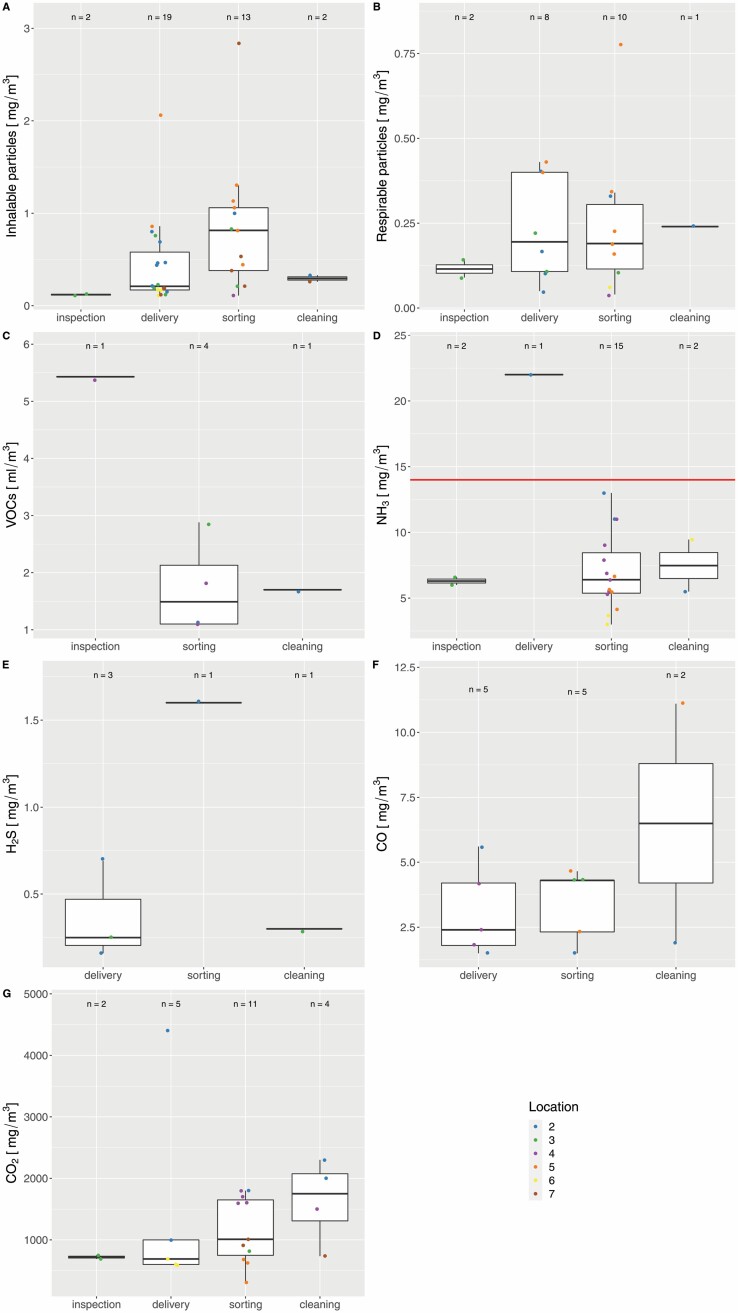
Boxplots for averaged concentrations of (A) inhalable particles, (B) respirable particles, (C) VOCs, (D) ammonia, (E) hydrogen sulphide, (F) carbon monoxide, and (G) carbon dioxide. Values are colour coded for their measured location and artificially categorized to workplace categories. The red line depicts the occupational exposure limit value according to German TRGS 900 (The German Committee on Hazardous Substances, 2006). At location 1, no hazardous substances were measured.

For all hazardous substances, the shift average concentrations were determined (Fig. 1). Generally, the inhalable particle fraction was measured in the range between 0.11 and 1.3 mg m^−3^ (Fig. 1A) and the respirable particle fraction between 0.04 and 0.43 mg m^−3^ (Fig. 1B). Highest values have been determined for both particle fractions in the delivery category with 2.06 and 0.43 mg m^−3^ and the sorting category with 2.84 and 0.78 mg m^−3^, respectively. The concentration of VOCs was ranging from 1 to 5.5 ml m^−3^ (Fig. 1C). However, due to the low concentration in the initial measurements, VOCs have been monitored only in rare cases. The majority of the shift average values for ammonia ranged from 3 to 13 mg m^−3^ (Fig. 1D). The sole measured value in the delivery category was above the OELV of 14 mg m^−3^ (The Commission of the European Communities, 2000). Compared with the other investigated substances, the measured concentrations of ammonia were the highest in relation to the corresponding OELV. Concentrations of hydrogen sulphide were in the range of 0.16 to 1.6 mg m^−3^ (Fig. 1E), with an OEL of 7 mg m^−3^ (The European Commission, 2009). Also the measured concentrations for carbon monoxide (Fig. 1F) and carbon dioxide (Fig. 1G) were below their respective national OELV (The German Committee on Hazardous Substances, 2006), with 35 and 9100 mg m^−3^ and EU-wide OELV with 23 and 9000 mg m^−3^, respectively (The Commission of the European Communities, 2006; The European Commission, 2017). Cobalt and selenium, which may be utilized as supplements for fermentation, were below their limit of quantitation in all analysed samples (data not shown).

### Multiple increases of biological agent parameters inside biogas plants

**Figure 2.**
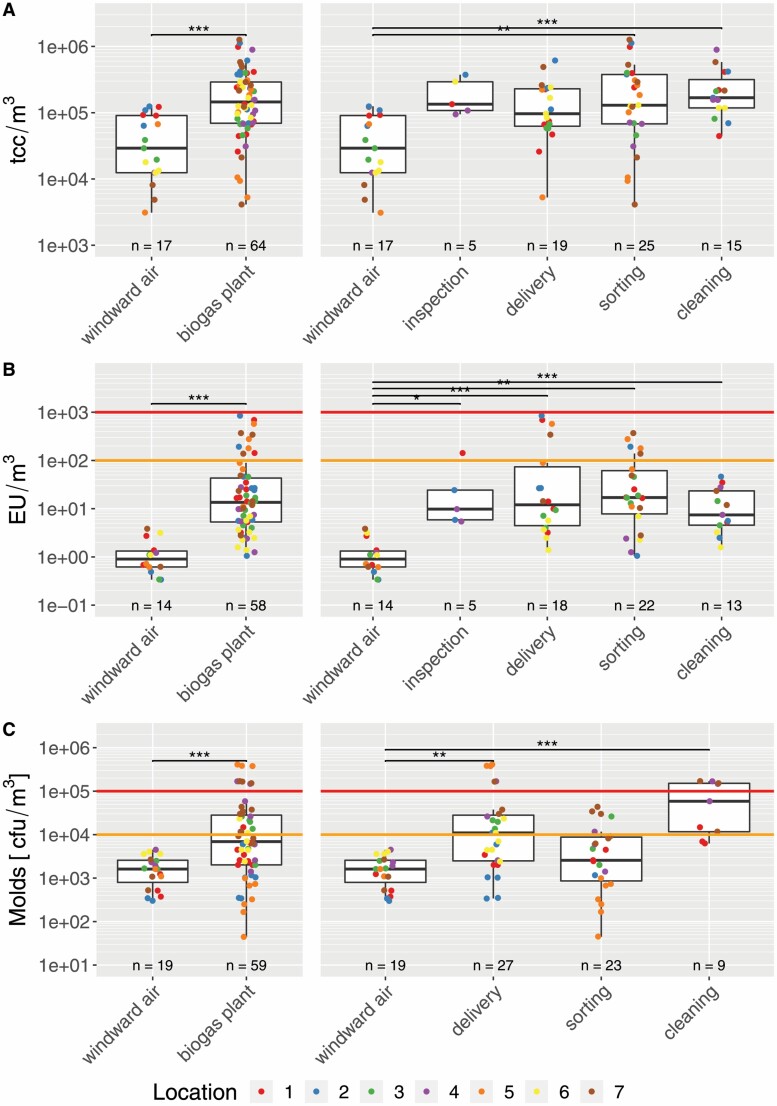
Boxplots of (A) total cell count, (B) endotoxins, and (C) moulds with the results of Wilcoxon tests (left) and Dunn tests depicted as black horizontal lines with asterisks indicating the *P*-value (right panels as *post hoc* test after Kruskal–Wallis; *P < 0.05, **P < 0.01, and ***P < 0.001). Indoor versus windward air comparisons are depicted on the left side of each plot, whereas categorical comparisons are on the right side. Values are colour coded for their measured location. Orange and red lines depict thresholds for endotoxins and mould exposure levels according to German TRBA 400 (The German Committee on Biological Agents, 2017).

The quantitative analyses of the total cell count (Fig. 2A), endotoxins (Fig. 2B), and moulds (Fig. 2C) were compared: first, inside the biogas plant and windward air (Fig. 2, left) and furthermore each workplace category to the windward air (Fig. 2, right).

The median of the total cell count per cubic metre increased about fivefold from ~3 × 10^4^ TCC m^−3^ outside of the biogas plant to ~1.5 × 10^5^ TCC m^−3^ inside the biogas plant (Fig. 2A, left plot). The range in the windward air comprised nearly two orders of magnitude from 3 × 10^3^ TCC m^−3^ to 1.25 × 10^5^ TCC m^−3^, inside the biogas plant the total cell count comprised a concentration from 4 × 10^3^ TCC m^−3^ to 1.25 × 10^6^ TCC m^−3^, i.e. nearly three orders of magnitude. The different categories showed an approximate fivefold increase of the medians (3.3- to 5.8-fold) in relation to windward air (Fig. 2A, right plot). The category ‘delivery’ and ‘cleaning’ showed highest TCC m^−3^ but also with a range of two to three orders of magnitude.

The median of the concentration of endotoxins increased inside the biogas plant about 15-fold from 0.9 to 13.5 EU m^−3^ (Fig. 2B, left plot). The range was from 0.3 to 4 EU m^−3^ for the windward air and from 1 to 850 EU m^−3^ inside the biogas plant.(The German Committee on Biological Agents, 2017) The increase in the median of the endotoxins differed slightly between the various categories ranging from 8- to 19-fold (Fig. 2B, right plot). In total, 10 of 54 samples were within the ‘increased’ exposure level of the TRBA 400 (The German Committee on Biological Agents, 2017). Highest absolute concentrations and medians were found again in the delivery and sorting category, but the medians of each category were approximately at 10 EU m^−3^ and thus 10-fold below the ‘increased’ exposure level of the TRBA 400.

In windward air, the concentration of the sum parameter mould varied approximately one order of magnitude: 3 × 10^2^ to 4.5 × 10^3^ cfu m^−3^ (Fig. 2C, left plot). Inside the biogas plant, the concentration spread from 4 × 10^1^ to 4 × 10^5^ cfu m^−3^ corresponding to four orders of magnitude In general, the median of moulds increased inside the biogas plant by a factor of 4.3 compared with windward air. The cleaning category revealed highest concentrations with a median 36 times higher than the windward air median (Fig. 2C, right plot). Inside the biogas plant, 58 samples were taken and 25 were in the ‘increased’ exposure level and 9 samples were in the ‘high’ exposure level according to TRBA 400. Also, the medians of the delivery and cleaning category were in the ‘increased’ range. It is noteworthy, that nine samples were taken in the cleaning category and seven samples were in the ‘increased’ range and four samples in the ‘high’ range of the TRBA 400.

**Figure 3.**
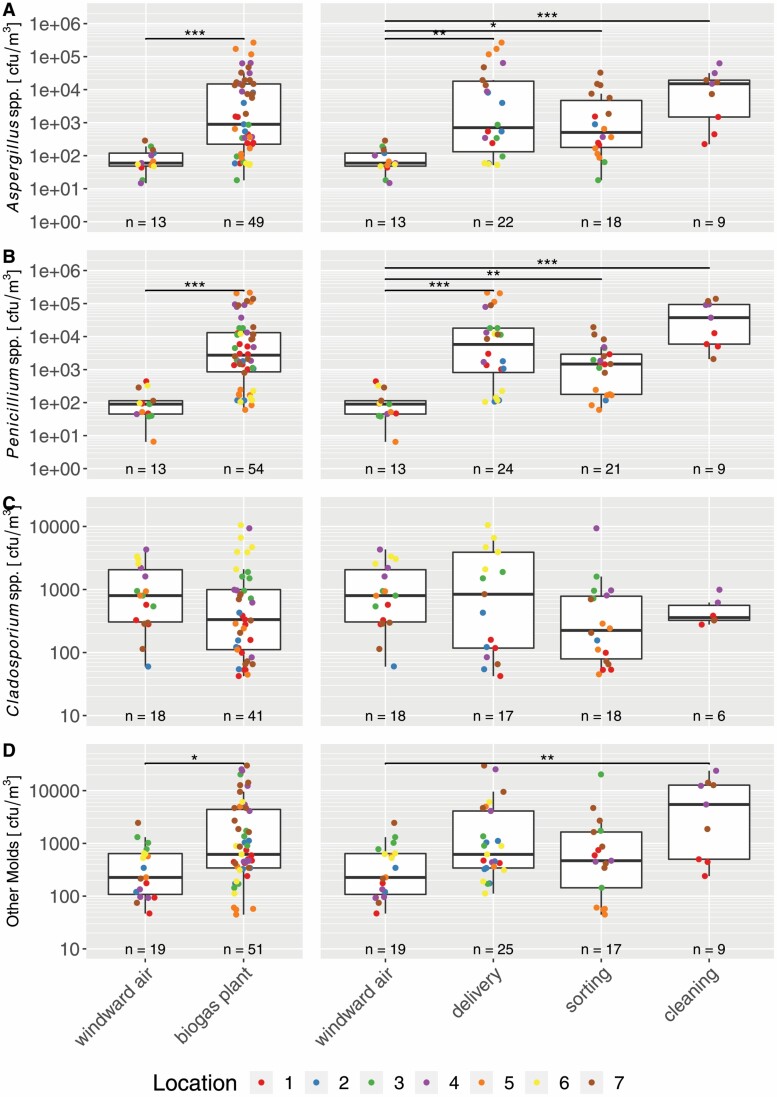
Boxplots of (A) *Aspergillus* spp., (B) *Penicillium* spp., (C) *Cladosporium* spp., and (D) other moulds with results of Wilcoxon tests (left) and Dunn tests depicted as black horizontal lines with asterisks indicating the *P*-value (right panels as *post hoc* test after Kruskal–Wallis; *P < 0.05, **P < 0.01, and ***P < 0.001). Indoor versus windward air comparisons are depicted on the left side of each plot, whereas categorical comparisons are on the right side. Values are colour coded for their measured location.

As moulds were identified down to the species level, analyses were split up for the following genera, representing the most abundant genera in the analysed samples: *Aspergillus* spp. (Fig. 3A), *Penicillium* spp. (Fig. 3B), and *Cladosporium* spp. (Fig. 3C). The remaining moulds were reanalysed as well (Fig. 3D).

Outside the biogas plant, the concentration of *Aspergillus* spp. ranged from 14 to 290 cfu m^−3^, whereas inside the biogas plant, it ranged from 5 × 10^1^ to 2.6 × 10^5^ cfu m^−3^ (Fig. 3A, left plot). The median of *Aspergillus* spp. increased by a factor of 15 inside the biogas plant compared with the windward air. Comparing the categories to the windward air showed an increase of 250-fold in the cleaning category (Fig. 3A, right plot), 9-fold for sorting, and 12-fold for delivery. The increases for *Penicillium* spp. were similar (Fig. 3B, left plot). Outside the biogas plant, this genus ranged from 6 to 440 cfu m^−3^, inside the biogas plant from 6 × 10^1^ to 2.1 × 10^5^ cfu m^−3^, representing an increase in the median by a factor of 30. For the different categories, the increase was 16-fold in sorting, 64-fold in delivery, and 415-fold in the cleaning category (Fig. 3B, right plot). These findings are significant for each workplace category, i.e. delivery, sorting, and cleaning. Also, the remaining moulds increased inside the biogas plant (Fig. 3D, left plot). On the contrary, the median of concentrations of *Cladosporium* spp. decreased in the biogas plant by approximately twofold without being significant (Fig. 3C, left plot), which could also be found when comparing the different workplace categories (Fig. 3C, right plot).

**Figure 4.**
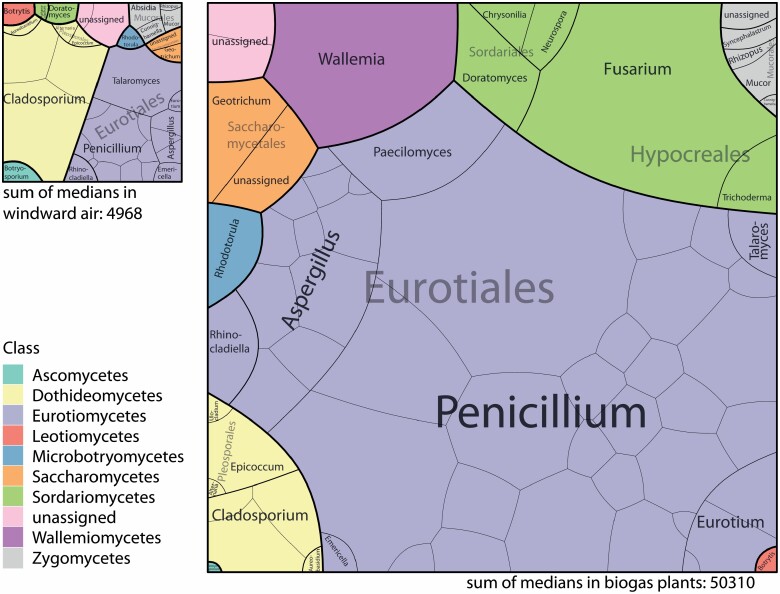
Treemap of each analysed mould species representing the cfu m^−3^ as area in windward air (left plot) and inside the biogas plant (right plot) with the sum of medians below the plot. Taxonomic levels are separated by different colours and line thicknesses as given in the legend. For purposes of readability, the species names were omitted in each box.

**Figure 5.**
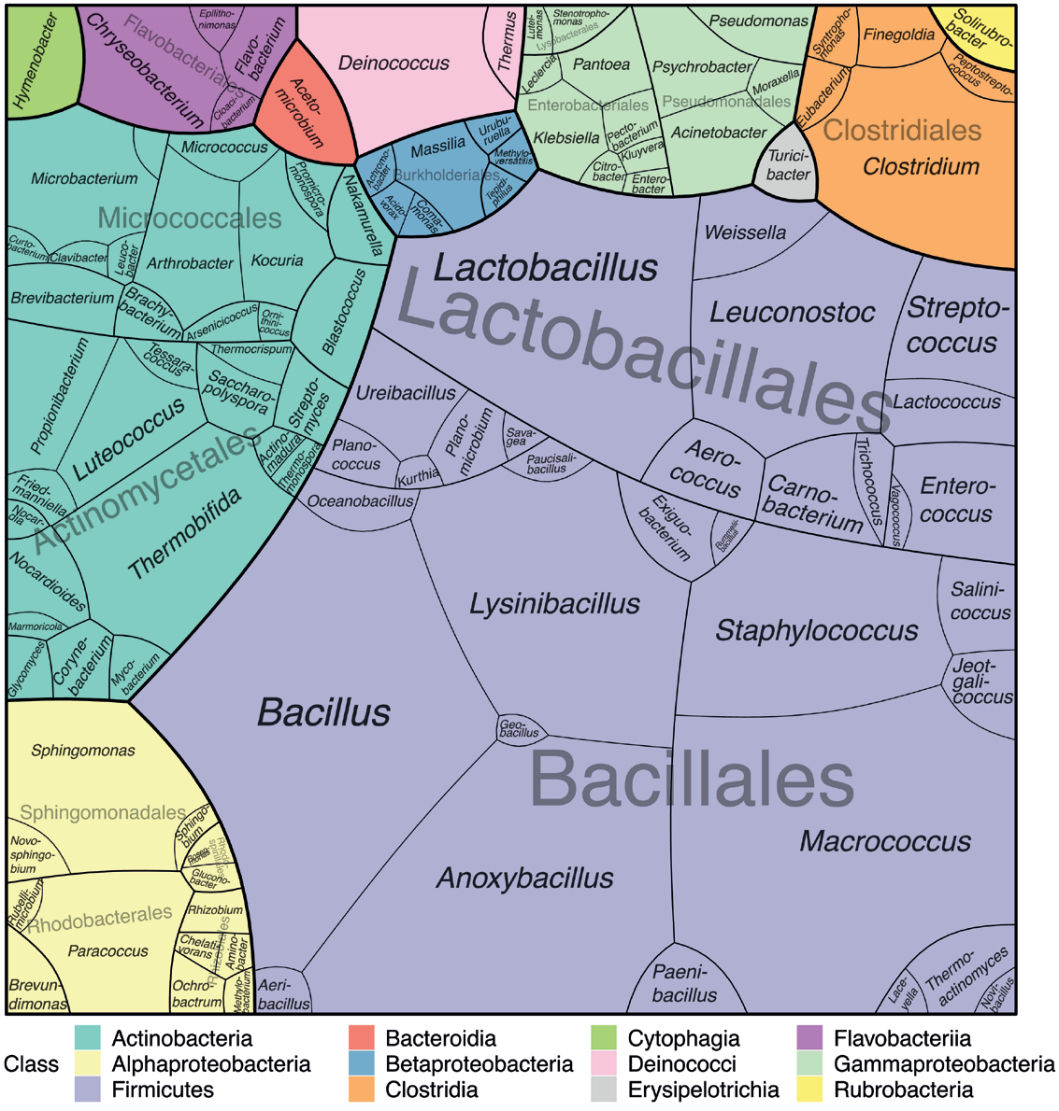
Treemap of each analysed bacterial species representing the fraction of analysed clones in the clone library in biogas plants. Taxonomic levels are separated by different colours and line thicknesses as given in the legend.

The complete mould composition was visualized by treemaps for outdoor air (Supplementary Fig. S1, available at *Annals of Work Exposures and Health* online) and biogas plants (Fig. 4). The voronoi treemap aims to fill a given area utilizing quantitative data on different levels that are dependent on each other. In this case, areas were calculated from median values for each mould species inside and outside the biogas plant. Subsequently, areas for each genus were calculated by summing up the values of the species belonging to the genus. Then the same was done for the taxonomic family, order, and class. As the sum of all medians inside the biogas plant (50 310) was larger than the sum in windward air (4968), the total area was ~10-fold larger (Fig. 4). As a side note due to the different approaches on calculating the median on species level (Fig. 5) and on the kingdom level (Fig. 2C), there are differences in the median values. For example, in windward air, the median on kingdom level is 1616 cfu m^−3^ and summing up the medians on species level gives a value of 4968 cfu m^−3^. By comparing the treemaps for windward air and the biogas plant bioaeosols, the area of *Cladosporium* hardly changed, whereas the area of the Eurotiales, representing also *Aspergillus* and *Penicillium* increased dramatically, which was already shown in the boxplots is here visualized in the context of the remaining moulds; as an example, *Wallemia* spp. was not analysed in windward air but with ~7% of all moulds inside the biogas plant.

Analysing the bacterial composition via clone libraries does not allow absolute quantitation of bacterial quantities, but their fraction in the clone library can be relatively quantified. In Fig. 5, these fractions are visualized in a treemap, omitting unassigned sequences that represent 38% of the sequences. Furthermore, the species level was also omitted as not all sequences resolved down to species level. These results give a good overview on the bacterial composition of bioaerosols in biogas plants. Sixty-two percent of the assigned sequences (i.e. ~38% of all sequences) represent the class Bacilli with the orders of Bacillales (45%) and Lactobacillales (17%) and 17% the class Actinobacteria with the orders Actinomycetales (10%) and Micrococcales (7%). The most prominent species are *Bacillus* (10.4%) and *Macrococcus* (9.8%). Unfortunately, up to now the bacterial composition of the windward air could not be disclosed, which is a result of the minimal DNA concentration in this kind of bioaerosol.

## Discussion

The results for hazardous substances show relatively narrow variances throughout the different workplace categories, at least for the most relevant parameters with several repetitions, i.e. inhalable and respirable particles or ammonia. Thus, these results give a first impression about hazardous substances in biogas plants. On the contrary, the results for biological agents spread for each parameter across several orders of magnitude. Based on the results, generalization of the exposure to biological agents in biogas plants is hardly possible. Selected analyses of specific workplaces are preferable as recommended by risk assessment policies.

In general, the analyses of hazardous substances were unremarkable. With the exception of ammonia, all measured concentrations were significantly lower than the corresponding OELVs. For ammonia, a single measured concentration exceeded the OELV in the working area of the delivery. As this working area is not a permanent workplace, it is likely that workers are not exposed during the whole shift. However, short-term exposure may be relevant. Hence, technical measures like ventilation or personal protective equipment should be adapted to this working area.

Traversi *et al.* (2018) did a similar approach in biogas plants and compared their results with other studies at different occupational settings. For endotoxins, they found a bandwidth of two orders of magnitude ranging from 0 to 138 EU m^−3^ for the PM_4.5_ fraction. In our analyses, the endotoxin concentration ranged from 1 to 840 EU m^−3^, i.e. nearly three orders of magnitude. However, compared with other studies, we analysed the inhalable particle fraction according to European Standard EN481 (Deutsches Institut fur Normung, 1993) including also particles having an aerodynamic diameter larger than 4.5 µm. This may explain the higher concentrations. Classification of the values into an occupational health context is difficult as different countries propose various limits: (Health Council of the Netherlands, 2010) recommends a ‘health-based recommended occupational exposure limit’ of 90 EU m^−3^ as average across an 8 h workshift. The German Committee on Biological Agents defines different exposure limits with relation to the exposure duration and frequency. The lowest level ‘increased’ comprises concentrations between 100 and 1000 EU m^−3^. Focussing on the windward air samples, values in this study ranged from 0.4 to 3.8 EU m^−3^, thereby resembling nicely values from other ambient air studies (Rolph *et al.*, 2018).

Traversi *et al.* (2018) observed concentrations up to 26 000 cfu m^−3^ of moulds (mean 4800 cfu m^−3^), but the analysis was limited to one growth condition (Traversi *et al.*, 2018). In contrast, we have analysed molds on two different media and at two temperatures and detected a range of 45–400 000 cfu m^−3^, which resemble previously published results (Traversi *et al.*, 2018). Nonetheless, the mean was at ~7000 cfu m^−3^ and the interquartile range from 2000 to 30 000. Thus, the middle-positioned values of our study support the results of previous studies. Comparing the mould concentrations to exposure limits of the TRBA 400, we found that 40% of the samples were in the lowest level ranging from 1 × 10^4^ to 1 × 10^5^ cfu m^−3^ and 15% were even in the mid-level ‘high’ ranging from 1 × 10^5^ to 1 × 10^6^ cfu m^−3^. Taken together, the results of moulds and endotoxins in comparison to the TRBA 400 and the statement that the analysed workplaces are no permanent posts, one can conclude that the exposure is rather low at these workplaces.

In addition, moulds were identified by their macroscopic and microscopic characteristics. We found a drastic change in the mould composition in biogas plants compared to the outdoor air with a 200- to 400-fold increase in concentrations of the genera of *Aspergillus* and *Penicillium*. A similar increase was found for the paper recycling industry as well (Klug *et al.*, 2011; Degois *et al.*, 2017). The quite constant concentration of *Cladosporium* spp. was also detectable in both industries, as well as an increase in concentrations of members of the genus *Eurotium*. These findings suggest a revision of the sum parameter ‘moulds’ for risk assessment targeting biological agents because the general increase in moulds does not reflect the workplace-specific increase, e.g., in *Aspergillus* spp. The various identified *Aspergillus* species comprised *Aspergillus flavus*, *Aspergillus fumigatus* and the complex of *Aspergillus niger*. These species incorporate an infectious, sensitizing, as well as toxic potential, and their increase across several orders of magnitude at various workplaces should be reflected in the risk assessment. Assessing specific organisms rather than sum parameters was also suggested by Walser *et al.* (2015).

Interestingly, the similarity between biogas plants and paper recycling industry was not only found in the composition of moulds, but also found in the composition of bacteria. Klug *et al.* (2011) found that genes, closely related to the members of the Firmicutes dominated in the clone library. This was also found by Degois *et al.* (2017) but the phylum of proteobacteria was even more prominent. However, compared with the outdoor reference, the concentration of proteobacteria was lower. Thus, a high concentration of firmicutes may be as characteristic as the increase of *Aspergillus* and *Penicillium* for bioaerosols in biogas plants.

It could be expected that the composition of hazardous substances and microorganisms inside the production pipeline, i.e. anaerobic digestion, digestate storage, or gas processing units, tremendously differs from the composition of these parameters at the workplaces. In the production pipeline, dominant gaseous hazardous substances are carbon dioxide with ~30%, methane with ~60%, and in lower concentrations hydrogen sulphide and ammonia based on several factors (Kuo and Dow, 2017; Laiq Ur Rehman *et al.*, 2019). Thus, nearly the complete gaseous content in the production pipeline consists of hazardous substances. Langer *et al.* (2019) investigated nine full-scale anaerobic digesters for their bacterial, archaeal, and fungal composition. In digesters with high methane yield, the bacterial communities were quite similar distributed with the order Clostridiales being highly abundant. Sequences closely related to *Clostridia* were also abundant in the analysed bioaerosols, but mainly sequences closely related to members of the order Bacillales or Lactobacillales were found. Nonetheless, *Lactobacillus* was also highly abundant inside a thermophilic digester and in ‘lab-scale’ reactors fed with cattle manure (Maus *et al.*, 2016; Campanaro *et al.*, 2018). Furthermore, Campanaro *et al.* (2018) found sequences closely related to *Bacillus*, *Microbacterium, Paracoccus*, *Arthrobacter*, *Streptococcus*, or *Enterococcus* in quite high abundancies. These studies support our findings of the bacterial composition in bioaerosols of biogas plants. Langer *et al.* (2019) found that the fungal composition inside the digester was dominated by sequences closely related to *Cladosporiaceae*, next to unassigned sequences. Next, abundant fungal families were *Microascaceae*, belonging to the class Sordariomycetes, or *Dipodascaceae* and *Piciaceae*, all belonging to the class of Saccharomycetes. At workplaces, bioaerosols were dominated by *Aspergillus* and *Penicillium* belonging to the class of Eurotiomycetes, followed by the class of Sordariomycetes and Dothideomycetes. The comparison of the analysed parameters at the workplace or inside the digester may be relevant during, e.g. opening of the production pipeline that can lead to a strong increase of methane (Fredenslund *et al.*, 2018). Furthermore, the subsequent removal, storage, and recycling of the digestate may be relevant at other, currently not analysed workplaces (Duan *et al.*, 2020). Nonetheless, it should be mentioned that the current risk assessment of further processing, e.g., of the digestate showed very low risks (Longhurst *et al.*, 2019).

In conclusion, we analysed hazardous substances and biological agents in workplace air of biogas plants. On the side of hazardous substances, only ammonia was found to be relevant in the range of the OELV; on the side of biological agents, we found an increase in all measured parameters, i.e. endotoxins, total cell count, fungi compared with windward air. However, the increase is rather low compared with workplaces in other industries, e.g. intensive farming. Remarkable is the change in the composition of fungi and the quite similar composition in other industries based on organic mass, e.g. paper recycling.

## Supplementary Material

wxab036_suppl_Supplementary_Figure_S1

wxab036_suppl_Supplementary_Table
