# Supplementary figures and images for: Multiplexed Workplace Measurements in Biogas Plants Reveal Compositional Changes in Aerosol Properties
Source: Ann Work Expo Health. 2021 Jul 5;65(9):1061–74. doi: 10.1093/annweh/wxab036 (PMC8577234; doi:10.1093/annweh/wxab036)

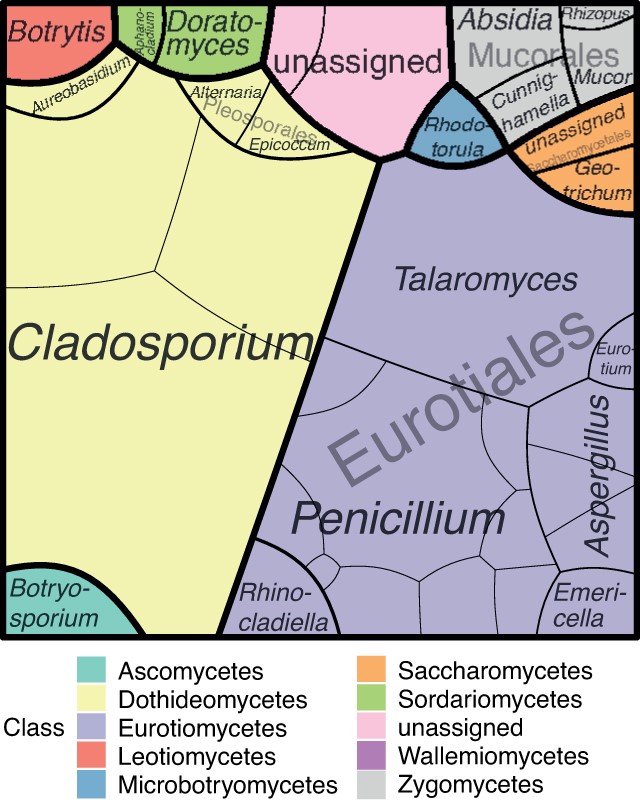

Supplement: wxab036_suppl_Supplementary_Figure_S1 [file wxab036_suppl_supplementary_figure_s1.jpeg]
